# Submarine canyon systems focusing sub-surface fluid in the Canterbury Basin, South Island, New Zealand

**DOI:** 10.1038/s41598-021-96574-3

**Published:** 2021-08-20

**Authors:** Priyadarshi Chinmoy Kumar, Tiago M. Alves, Kalachand Sain

**Affiliations:** 1 Wadia Institute of Himalayan Geology (WIHG), 33 GMS Road, Dehradun, Uttarakhand India; 2 Seismic Interpretation Laboratory, WIHG, Dehradun, Uttarakhand India; 3 3D Seismic Laboratory, School of Earth and Environmental Sciences, Cardiff University, Cardiff, UK

**Keywords:** Geology, Geophysics, Sedimentology, Tectonics

## Abstract

This work uses a high-quality 3D seismic volume from offshore Canterbury Basin, New Zealand, to investigate how submarine canyon systems can focus sub-surface fluid. The seismic volume was structurally conditioned to improve the contrast in seismic reflections, preserving their lateral continuity. It reveals multiple pockmarks, eroded gullies and intra-slope lobe complexes occurring in association with the Waitaki Submarine Canyon. Pockmarks are densely clustered on the northern bank of the canyon and occur at a water depth of 500–900 m. In parallel, near-seafloor strata contain channel-fill deposits, channel lobes, meandering channel belts and overbank sediments deposited downslope of the submarine canyon. We propose that subsurface fluid migrates from relatively deep Cretaceous strata through shallow channel-fill deposits and lobes to latter seep out through the canyon and associated gullies. The new, reprocessed Fluid Cube meta-attribute confirms that fluids have seeped out through the eroded walls of the Waitaki Canyon, with such a seepage generating seafloor depressions in its northern bank. Our findings stress the importance of shallow reservoirs (channel-fill deposits and lobes) as potential repositories for fluid, hydrocarbons, or geothermal energy on continental margins across the world.

## Introduction

Submarine canyons are features capable of eroding continental slopes^1^ to form principal conduits for sediment transported into deep-water environments^2–6^. They comprise steep-walled sinuous valleys with V-shaped cross-sections^2^ and their heads develop at the edge of the continental shelf. They eventually transition into fully developed channels with U-shaped cross-sectional profiles further downslope^2,7^. Submarine canyons occur on any type of continental margins—divergent, convergent or transform. On divergent margins their morphology is chiefly controlled by erosional and depositional processes^8^, whereas on convergent and transform margins they reflect a close interplay between tectonic and magmatic processes^9,10^. Hence, the origin of submarine canyons has consistently been attributed to slope erosional processes and the effect of cross-slope turbidity currents^2,3^.

The recent decade has witnessed multiple attempts at investigating the geomorphology of submarine canyons and channel systems, mostly due to their economic and environmental importance. For instance, the lower stretches of submarine canyons are associated with sediment-fan and lobe complexes hosting reservoir successions with economic importance^11^. In parallel, canyon heads are preferential sites for benthic habitats^12^, pelagic and demersal fishing stocks, also comprising feeding grounds for Cetaceans^13^. When such submarine canyons are filled with sand-rich sediments, they serve as prolific geological reservoirs^3,14^, while being linked to natural fluid seeps^15^. In parallel, turbidite and mass-flows transported through canyons can break communication cables on the seafloor^16^ and damage subsea infrastructure^17^.

**Figure 1:**
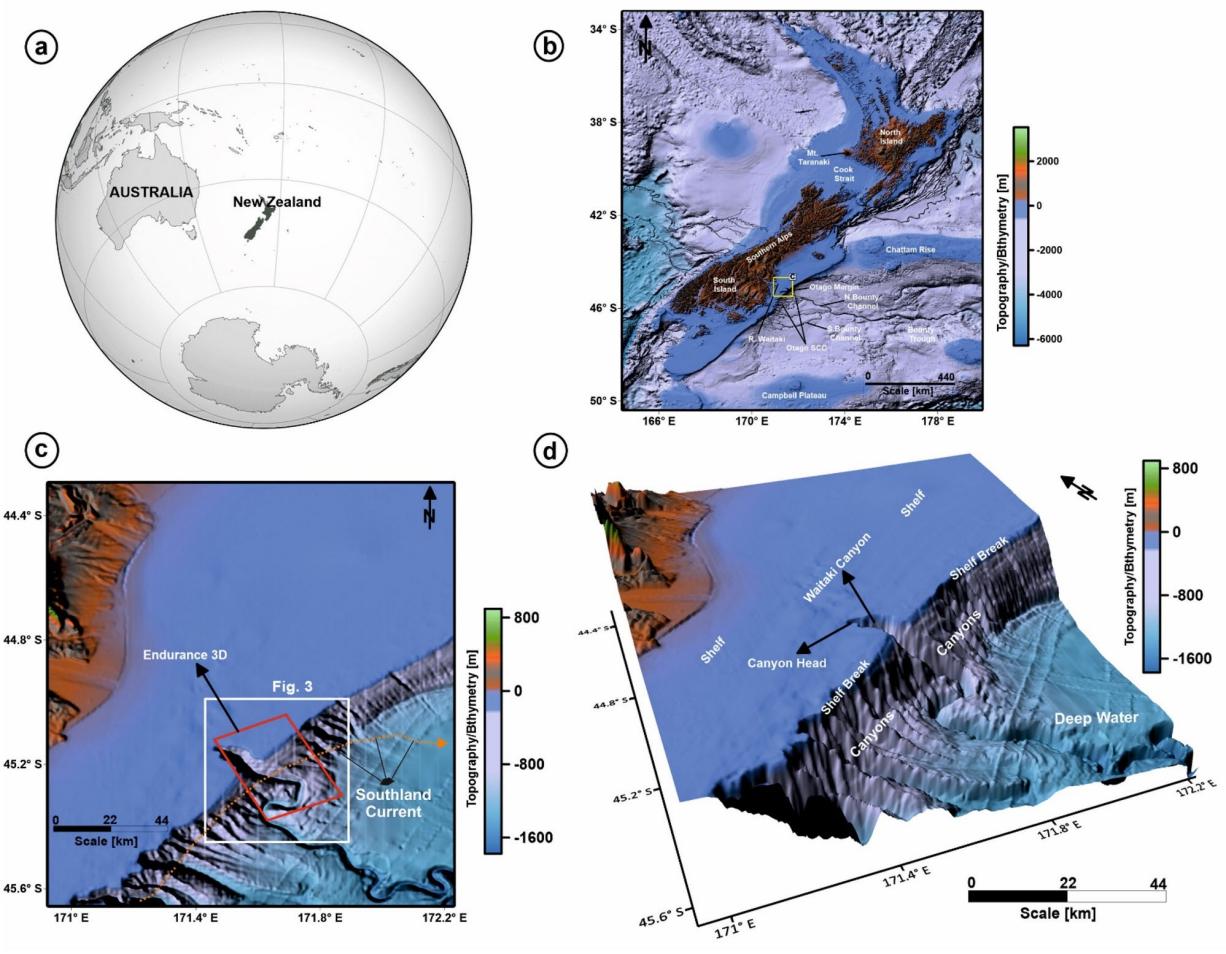
(**a**–**d**) Canterbury Basin, located offshore New Zealand to the east of South Island. The southeast margin of the South Island contains a network of submarine canyons, the so-called Otago Submarine Canyon Complex (OSCC), which incises the Otago shelf. The Waitaki Submarine Canyon lies to the northern part of the continental shelf and is incised at the shelf-break, with its head trending towards the shelf. The location of the Endurance 3D seismic volume is highlighted by a red rectangular box in (**c**) around the Waitaki Submarine Canyon. The flow of the Southland Current is indicated by the orange dashed curve. The present-day Waitaki Submarine Canyon receives terrigenous sediments from the continental shelf that is distributed via rivers and canyons throughout the Bounty Trough. (The above maps are prepared using licensed version of Corel Draw v16 and OpendTect interpretation Suite v6.6.3).

The Waitaki Submarine Canyon, a shelf-incised canyon that forms part of the Otago Submarine Canyon Complex, is located along the southeast margin of the South Island of New Zealand, in the so-called Canterbury Basin (Fig. 1b–d). At present, the Waitaki Submarine Canyon receives terrigenous sediment from the continental shelf, rivers and sub-aerial canyons of New Zealand^18–20^. Previous research^20–22^ was limited to studying the physiography and significance of seafloor depressions in the vicinity of canyons formed on the Otago Shelf and Central Chatham Rise using 2D seismic profiles and multibeam bathymetric maps. Associated turbidite depositional systems remain poorly documented, though sandy canyon-fill deposits represent the most promising targets for hydrocarbon and geothermal exploration in the Canterbury Basin^14,23,24^. In summary, this research aims to:

- Decipher the depositional architecture and structure of near-seafloor strata around the Waitaki Submarine Canyon using high-quality 3D seismic data;
- Investigate the role of submarine canyons in controlling continental-margin architecture and sedimentary processes;
- Discuss the importance of channel-fill deposits and lobes as capable of focusing fluid flow in deep-offshore basins.

## Geological setting

### Tectonic evolution of the Canterbury Basin

The eastern part of the South Island comprises a divergent continental margin on which the Canterbury Basin is located (Fig. 1a). This basin lies over thinned continental crust of a discrete tectonic plate (Zealandia) that has broken away from Antarctica and Australia around 80 Ma^25^. The depositional history of the Canterbury Basin has, since then, been controlled by the uplift and erosion of the Southern Alps, marine currents and sea-level change^18,20^. As a result, the basin extends, at present, from a relatively wide continental shelf (~ 100 km) with a depth ranging ~ 140–145 m to a maximum water depth of ~ 1500 m^26^.

**Figure 2:**
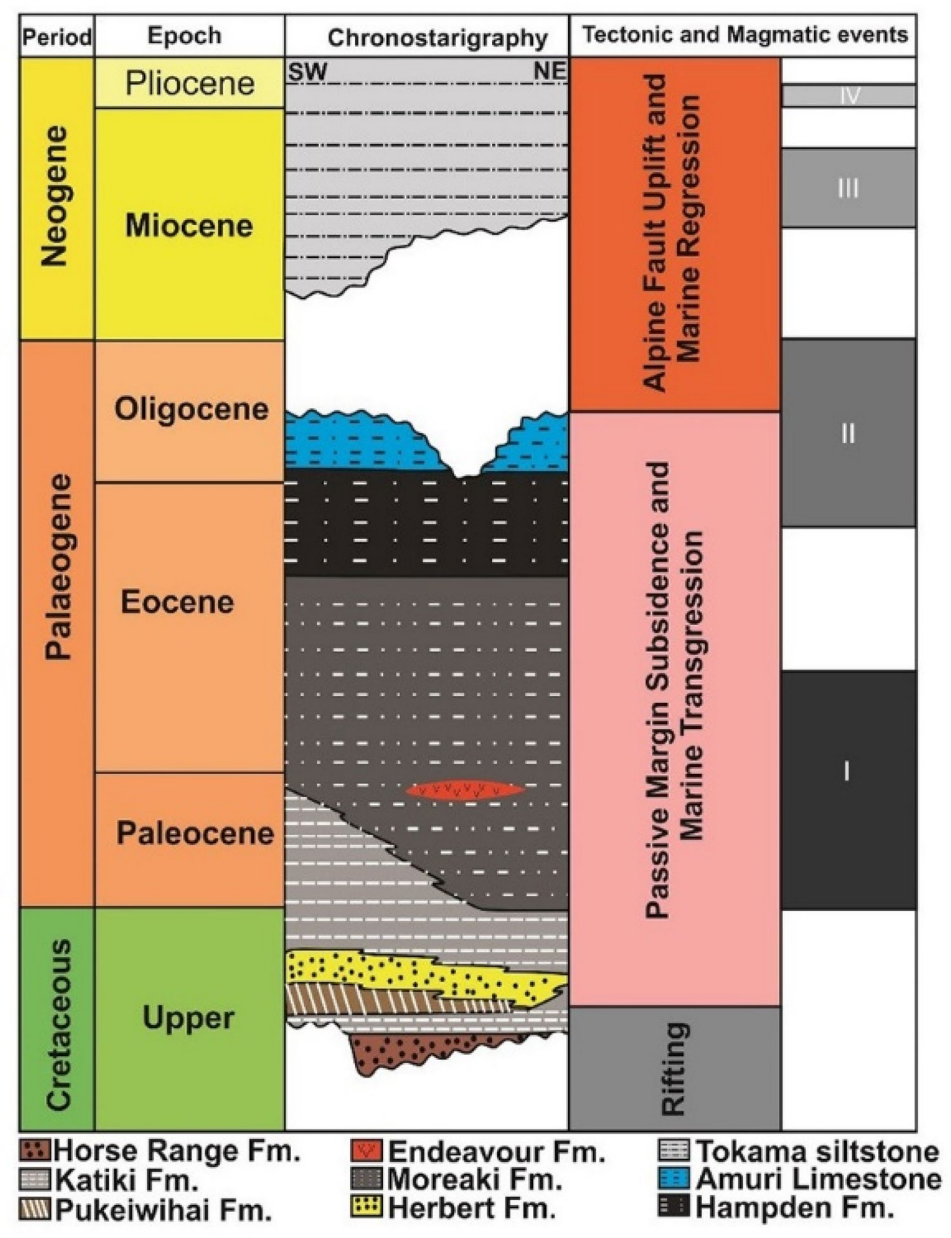
Lithostratigraphic framework of the Canterbury Basin, offshore New Zealand, based on Carter^27^ and Uruski^26^.

The Canterbury Basin covers a total area of ~ 50,000 km^2^ and is bounded by the Chatham Rise and Bounty Trough (Fig. 1). Its basin fill includes sedimentary units ranging from the Cretaceous to the Holocene in age. Basement rocks consist of the Torlesse Supergroup, a unit dominated by alternating metasediments (greywacke and argillites) of Permian to Early Cretaceous ages^26,27^. Cretaceous continental rifting generated several E–W extensional basins, which were subsequently filled by fluvial and paralic sediments of the Horse Range and Katiki formations^26,27^ (Fig. 2).

The Late Cretaceous marks the onset of post-rift subsidence and subsequent marine transgression in the Canterbury Basin^27^. This led to a change in deposition from terrestrial sandstones and coal (Pukeiwihai Formation, Fig. 2) to marine conglomerates, sandstones, mudstones, siltstones and minor coal measures towards the end of the Cretaceous (Katiki, Moreaki and Hampden formations; Fig. 2). Late Eocene to Early Oligocene units include fine-grained carbonates of the Amuri Limestone, which mark the maximum extent of marine transgression in the Canterbury Basin. Above the Amuri Limestone occurs a regressive sequence.

An Oligocene maximum flooding surface comprises a current-induced stratigraphic surface named the *Marshall Paraconformity*^27,28^. This paraconformity is overlain by hemipelagic, bioclastic limestones and glauconitic greensands that mark a sharp decrease in the influx of terrigenous sediment into the basin. The Late Oligocene and Early Miocene witnessed important changes in regional tectonics, with uplift of the Alpine fault and erosion of the Southern Alps predominating from thereon in, promoting a rapid influx of terrigenous sediment (mostly siltstones) to the east^20^.

The Late Miocene to Recent is marked by the deposition of large, elongated clinoform drifts (contourites), forming the so-called *Canterbury Drift*^18,29^. These sediment drifts became ubiquitous over the SE New Zealand margin at the end of the Miocene and control the present-day architecture of strata in the Canterbury Basin. Sea-level change and a relative increase in the strength of marine currents acted as primary controls on sedimentation during the Late Quaternary^18^.

### Evolution of the Otago Submarine Canyon Complex

The Otago Submarine Canyon Complex comprises a set of submarine canyons and gullies that incise the continental shelf and slope of SE New Zealand (Fig. 1b). These canyons have channelised terrigenous sediment into the abyssal Bounty Trough and corresponding fan since the Early Miocene^18,20^. The shelf-slope break in this region is incised by this canyon complex, forming regularly-spaced canyon heads^19,20^.

**Figure 3:**
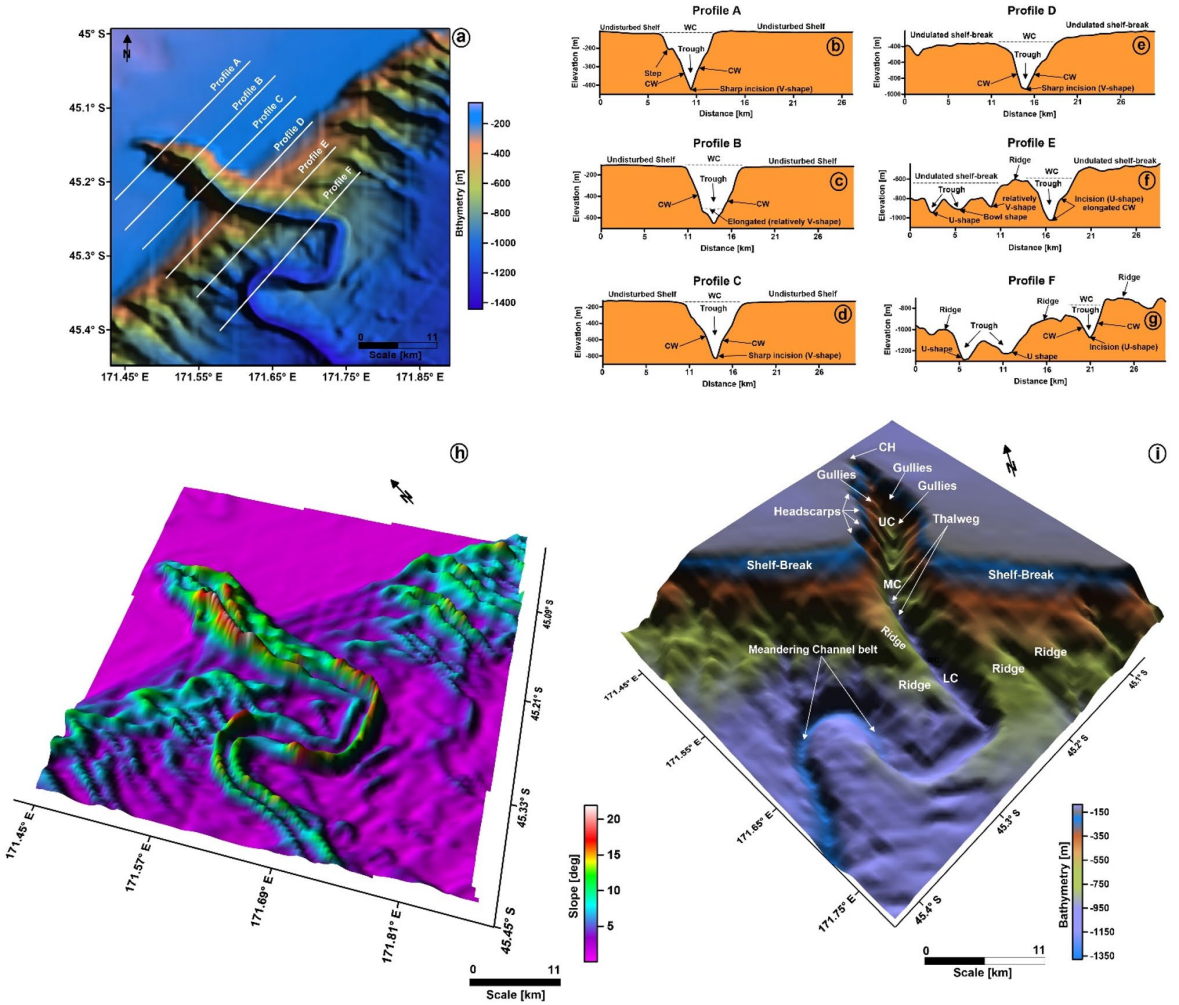
(**a**) Bathymetry map highlighting the geometry of the Waitaki Submarine Canyon at the shelf-break; (**b**–**g**) Profile cross-sections (A–F) along the dip of the canyon showing the nature of seafloor incisions made by the Waitaki Submarine Canyon at its upper course (Profiles A–C in **b**–**d**), middle course (Profiles D–E in **e**, **f**) and lower course (Profile F, in **g**); (**h**) Slope gradient map of the canyon zone. The canyon contains steep-sided walls that are deeply incised into the shelf and slope of the Otago margin; (**i**) Physiography of the Waitaki Submarine Canyon showing different structural elements. The canyon consists of three structural domains: the Upper Course (UC), the Middle Course (MC) and the Lower Course (LC). The UC of the canyon contains the canyon head (CH), headscarps and gullies. The MC of the canyon contains the thalweg. The LC contains the ridges and meandering channel belts. These structural configurations make the canyon to have a V-shaped cross-section in the UC that slowly changes into a U-shape cross-section in the MC and LC. (The above maps are prepared using licensed version of Corel Draw v16 and OpendTect interpretation Suite v6.6.3).

The Waitaki Submarine Canyon is located in the northern part of the Otago Submarine Canyon Complex (Figs. 1d, 3). The head of this canyon is narrow, steep and continues downslope into a transitional area where a sinuous profile is recorded. The Waitaki Submarine Canyon intersects the North Bounty Channel further downslope. In bathymetric data, numerous seafloor depressions have been identified at the seafloor in the vicinity of the Waitaki Submarine Canyon^19^. This same canyon has been interpreted as an active conduit for sediment since the earliest Pleistocene, and possibly existed as far in time as the early Pliocene^19^.

## Results

### Canyon physiography

Bathymetric data reveal a shelf-break incised by a network of regularly spaced, sub-parallel submarine canyons (Figs. 1c,d, 3a). Based on the classification scheme proposed by Jobe et al.^5^, the Waitaki Submarine Canyon comprises a Type I canyon that resembles a V-shaped profile in its proximal part (shelf edge), transitioning into a U-shaped profile towards its distal part. Bathymetric cross-sections clearly reveal gradual changes in geometry (profiles A–F, Fig. 3a–g). The Waitaki Submarine Canyon is located in water depths ranging from ~ 400 to 1200 m and shows a NW–SE orientation.

Profile A reveals a V-shaped cross-section where the canyon walls are deeply incised to a depth of ~ 400 m (Fig. 3b). Such a type of cross-section continues from the continental shelf to the shelf break (Fig. 3c–e; profiles B–D), showing a subsequent increase in the depth of incision from ~ 400 to 1000 m. Moving from the shelf break to the continental slope, the cross-section changes to a U-shaped geometry (Fig. 3f,g; profiles E,F), and the canyon’s incision depth increases to ~ 1200 m. The continental slope becomes wavy with alternate ridges and troughs. The head and the sidewalls of the Waitaki Submarine Canyon are steep (~ 10°–20°) on the upper continental slope. Downslope, the canyon gradually widens but its sidewalls remain steep (~ 10°–15°) (Fig. 3h).

The Waitaki Submarine Canyon is structured into three distinct courses: upper, middle and lower. The upper course spans the outer continental shelf and includes the canyon head, steep-walled gullies, and scarps (Fig. 3i). The middle course lies over the shelf break and comprises the channel thalweg. The lower course is located on the continental slope and is characterised by its low-relief sidewalls, further changing into a meandering channel belt—thereby giving rise to a canyon-channel belt system (Fig. 3i).

### Seabed morphology in seismic data

**Figure 4:**
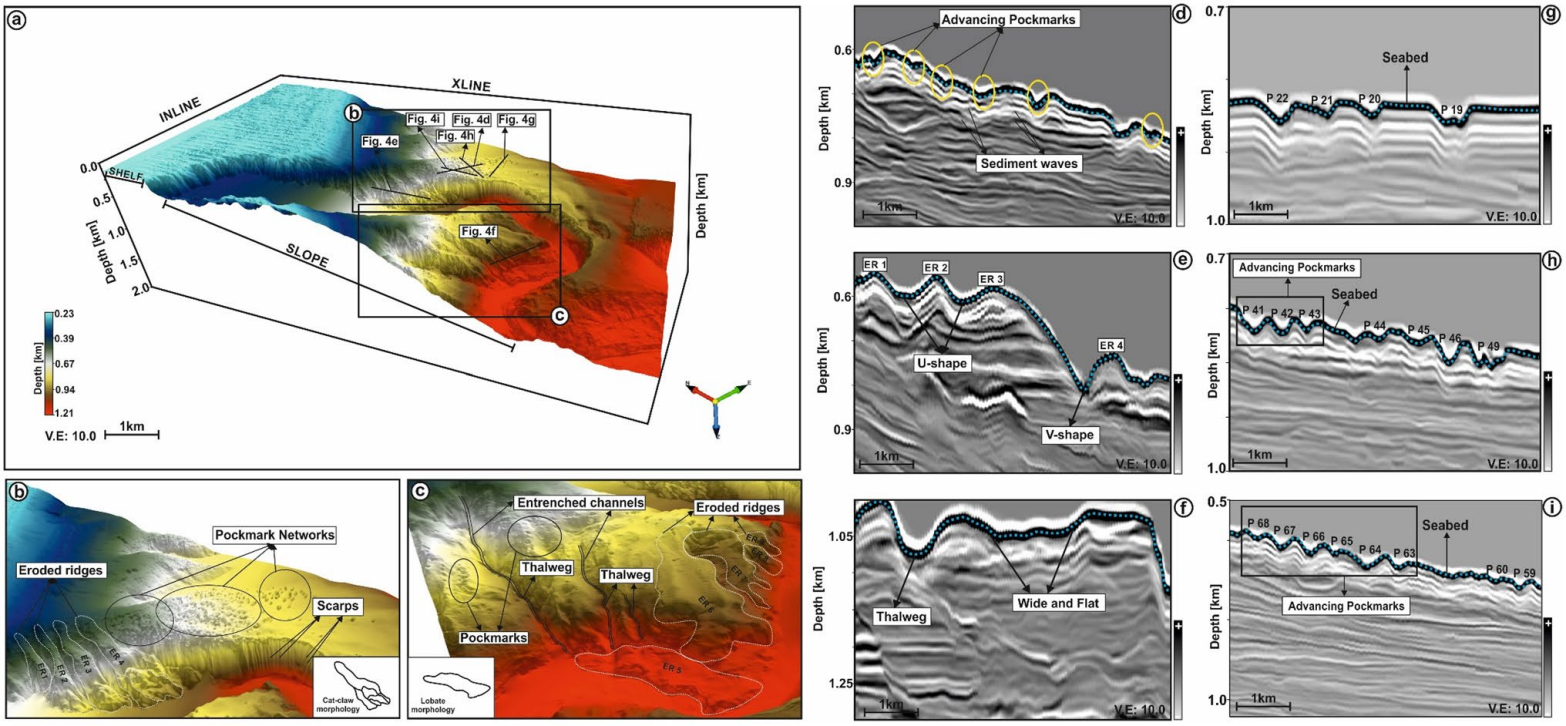
(**a**) TWT structure map of the seafloor. The seafloor is dissected by the Waitaki Submarine Canyon at the shelf break and further develops into a sinuous structure downslope; (**b**–**c**) Zoomed view of the NE and SW part of the seafloor. The NE portion of the seafloor is structured with eroded ridges (ER 1–4) and gullies with vertically stacked scarps. These eroded ridges show a distinct cat-claw morphology. Pockmarks are densely clustered over the northern ridge of the seafloor. In the SW portion of the seafloor, the eroded ridges (ER 5–9) show a distinct lobate morphology. Furthermore, the eroded gullies are entrenched in different channel belts; (**d**–**f**) Seismic profiles confirms the presence of these structures over the seafloor. Examples of mapped pockmarks displayed through seismic cross-sections (**g**–**i**). The pockmarks are associated with V-shaped depressions, which are stacked and closely spaced (typically known as advancing pockmarks). (The above maps are prepared using licensed version of Corel Draw v16 and OpendTect interpretation Suite v6.6.3).

Several eroded ridges (ER 1–ER 10) of a typical gullied slope succession are observed on the eastern and western flanks of the Waitaki Submarine Canyon (Fig. 4b,c). Ridges on the eastern flank (ER 1–ER 5) are deeply eroded, resembling a “cat claw” morphology downslope. Ridges show a maximum length and width of ~ 3945 and 1242 m for ER 4 and a minimum length and width of ~ 1887 and 585 m for ER 1. Ridge ER 4 covers a maximum area of 4.8 km^2^, whereas ER 1 reveals a minimum area of 1.02 km^2^ (Table 1).

**Table 1:** Morphometric parameters of the eroded ridges in the study area.

Name of eroded ridges (ER)	Length (m)	Width (m)	Aspect ratio	Area (km^2^)
ER 1	1887.46	584.85	3.22	1.02
ER 2	2997.82	914.67	3.27	2.13
ER 3	3166.95	941.02	3.36	2.51
ER 4	3945.01	1242.82	3.17	4.80
ER 5	5599.4	1312.29	4.26	5.94
ER 6	4856.16	2239.3	2.16	7.33
ER 7	3598.56	896.83	4.01	3.94
ER 8	1189.79	474.9	2.50	0.78
ER 9	911.19	492.48	1.85	0.42

The eroded ridges on the western flank (ER 5–ER 9) resemble a lobate morphology downslope. They show a maximum length and width of ~ 5599 and 1312 m for ER 5 and a minimum length and width of ~ 911 and 492 m for ER 9. ER 6 has a maximum area of 7.33 km^2^, whereas ER 9 is much smaller and reveals a minimum area of 0.42 km^2^ (Table 1). Tectonic forces uplifted the SW flank of the Waitaki Submarine Canyon and subsequently eroded it, thereby generating accommodation space for sediment routed through the middle and lower reaches of the incised canyon (Fig. 4a–c).

Seafloor pockmarks are observed on the NE bank and are mostly clustered over the ridges and gullies of the Waitaki Submarine Canyon. These depressions occur at a water depth of approximately 500–900 m (Fig. 4g–i). Pockmarks are closely spaced in cross-section and present a V-shaped morphology with crests and troughs (Fig. 4g–i). However, these structures are elliptical or circular in map-view (Fig. 4a).

**Figure 5:**
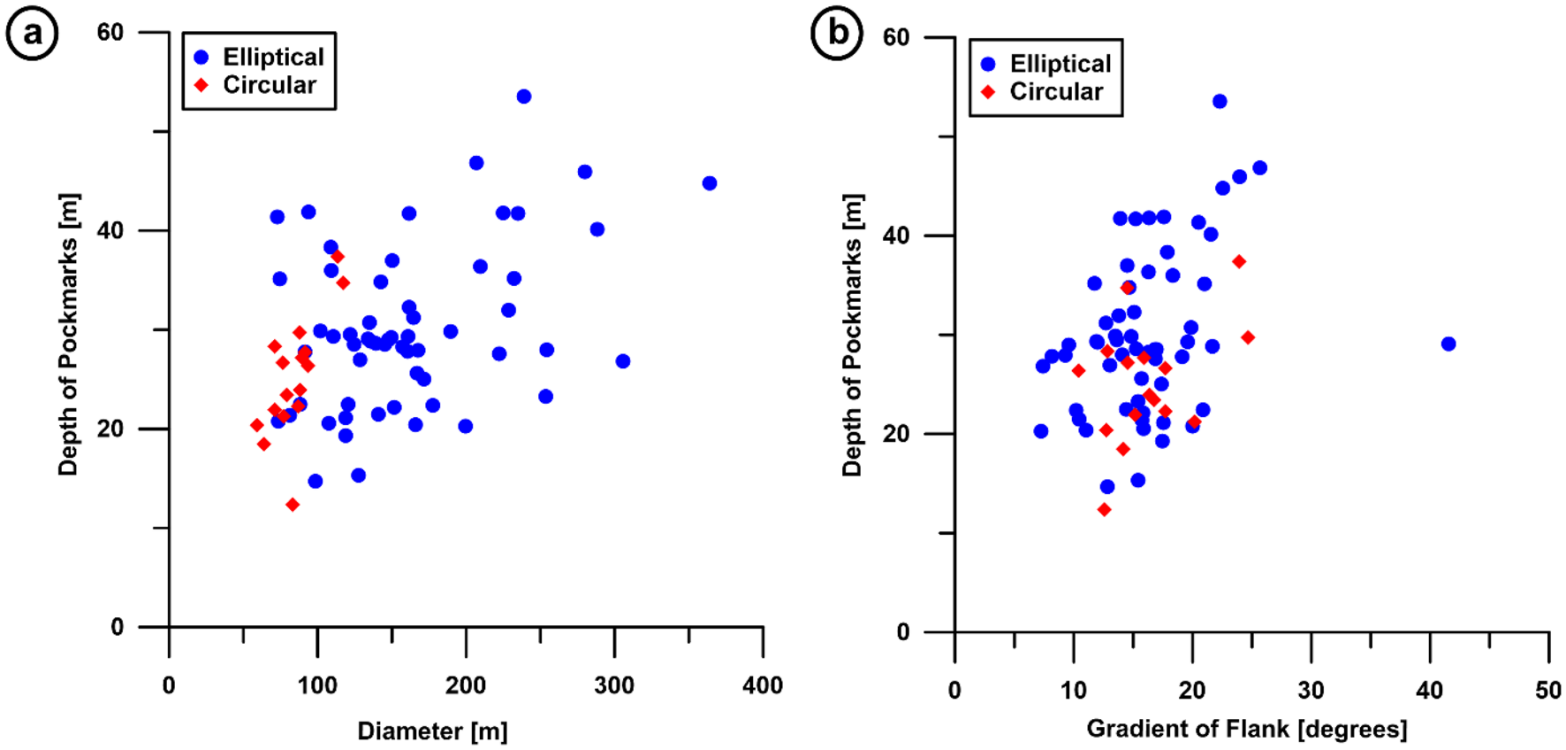
(**a**) Morphometric plot showing the depth of the pockmarks versus their diameter (n = 59 for elliptical pockmarks and n = 16 for circular pockmarks); (**b**) Morphometric plot showing the depth of the pockmarks versus gradient of their flanks (n = 59 for elliptical pockmarks and n = 16 for circular pockmarks).

On the seafloor, the depth of elliptical pockmarks varies between ~ 14.7 and 3.57 m (average of ~ 30.14 m) and diameter ranges from 72.78 to 364.08 m, for an average of ~ 161.95 m (Table 2; Fig. 5a). The depth of circular pockmarks varies between 12.39 and 37.38 m, for an average of 25.13 m, while their diameter ranges from 59.00 to 117.10 m (average of 84.30 m) (Table 2; Fig. 5a). The morphometric plot in Fig. 5a demonstrates that there is an overlap at 20–40 m when considering pockmark depth. When they reach these depths, the diameter of both circular and elliptical pockmarks varies between 50 and 100 m. However, the deepest pockmarks are usually elongated, giving rise to the elliptical sets more frequently observed on the seafloor.

**Table 2:** Morphometric parameters of the studied pockmarks.

Name of pockmarks	Long axis diameter (m)	Short axis diameter (m)	Average diameter (m)	Flank gradient (°)	Shape
P1	86.76	–	86.76	17.7	Circular
P2	117.1	–	117.1	14.51	Circular
P3	192.43	75.28	133.855	41.55	Elliptical
P4	146.28	57.118	101.699	13.515	Elliptical
P5	71.28	–	71.28	12.83	Circular
P6	214.02	84.7	149.36	12	Elliptical
P7	171.77	85.52	128.645	13.045	Elliptical
P8	207.35	70.96	139.155	15.245	Elliptical
P9	223.84	76.68	150.26	14.515	Elliptical
P10	159.73	57.7	108.715	17.885	Elliptical
P11	182.97	86.87	134.92	19.87	Elliptical
P12	76.37	–	76.37	17.685	Circular
P13	177.04	104.67	140.855	10.44	Elliptical
P14	195.29	99.93	147.61	9.585	Elliptical
P15	289.67	154.93	222.3	16.86	Elliptical
P16	187.31	141.77	164.54	12.695	Elliptical
P17	227.99	151.33	189.66	14.815	Elliptical
P18	93.53	–	93.53	10.395	Circular
P19	285.8	171.111	228.4555	13.78	Elliptical
P20	91.55	–	91.55	15.895	Circular
P21	228.154	105.36	166.757	15.73	Elliptical
P22	319.11	145.75	232.43	11.735	Elliptical
P23	311.99	194.95	253.47	15.4225	Elliptical
P24	342.89	127	234.945	15.185	Elliptical
P25	71.02	–	71.02	15.175	Circular
P26	59	–	59	12.755	Circular
P27	211.91	91.33	151.62	15.855	Elliptical
P28	355.02	95.07	225.045	16.32	Elliptical
P29	254.37	100.47	177.42	10.195	Elliptical
P30	210.49	121.53	166.01	11.04	Elliptical
P31	264.4	71.06	167.73	9.27	Elliptical
P32	215.1	106.39	160.745	8.145	Elliptical
P33	275.54	123.86	199.7	7.22	Elliptical
P34	216.811	106.5	161.6555	15.105	Elliptical
P35	88.12	–	88.12	16.375	Circular
P36	89.64	–	89.64	14.54	Circular
P37	113.46	–	113.46	23.92	Circular
P38	87.83	–	87.83	24.68	Circular
P39	63.71	–	63.71	14.145	Circular
P40	156.04	87.84	121.94	13.615	Elliptical
P41	217.46	67.87	142.665	14.655	Elliptical
P42	117.06	45.36	81.21	15.755	Elliptical
P43	228.04	124.58	176.31	15.135	Elliptical
P44	372.61	187.34	279.975	23.975	Elliptical
P45	349.13	159.47	254.3	14.105	Elliptical
P46	480.22	247.95	364.085	22.52	Elliptical
P47	384.04	192.27	288.155	21.55	Elliptical
P48	311.88	166.12	239	22.305	Elliptical
P49	472.42	139.107	305.7635	7.42	Elliptical
P50	266.69	152.44	209.565	16.3	Elliptical
P51	270.156	143.87	207.013	25.65	Elliptical
P52	158.4	59.9	109.15	18.345	Elliptical
P53	160.91	59.93	110.42	19.605	Elliptical
P54	107.45	39.66	73.555	19.985	Elliptical
P55	218.31	71.8	145.055	16.84	Elliptical
P56	123.83	52.32	88.075	14.415	Elliptical
P57	169.91	70.8	120.355	20.885	Elliptical
P58	77.04	–	77.04	20.19	Circular
P59	252.74	69.08	160.91	11.91	Elliptical
P60	177.9	70.95	124.425	16.945	Elliptical
P61	134.85	48.39	91.62	19.14	Elliptical
P62	176.47	60.94	118.705	17.45	Elliptical
P63	199.07	115.45	157.26	16.24	Elliptical
P64	79.23	–	79.23	16.755	Circular
P65	251.53	71.33	161.43	13.905	Elliptical
P66	120.36	28.71	74.535	21.005	Elliptical
P67	141.02	46.82	93.92	17.57	Elliptical
P68	114.82	30.74	72.78	20.5	Elliptical
P69	83.28	–	83.28	12.59	Circular
P70	177.55	37.73	107.64	15.87	Elliptical
P71	190.48	64.52	127.5	15.425	Elliptical
P72	158.31	38.71	98.51	12.821	Elliptical
P73	291.01	52.32	171.665	17.385	Elliptical
P74	219.21	52.65	135.93	21.655	Elliptical
P75	175.88	61.99	118.935	17.555	Elliptical

The gradient of the flanks of elliptical depressions fluctuates between 7.42° and 41.55°, for an average of 16.21° (Table 2; Fig. 5b). The flank gradient of circular pockmarks fluctuates between 10.39° and 24.68°, for an average value of 16.25° (Table 2; Fig. 5b). The morphometric plot between the depth and gradient of the flanks for both of the pockmarks shows a close overlap in flank-gradient values when pockmark depth reaches 20–40 m (Fig. 5b).

### Regional seismic stratigraphy

**Figure 6:**
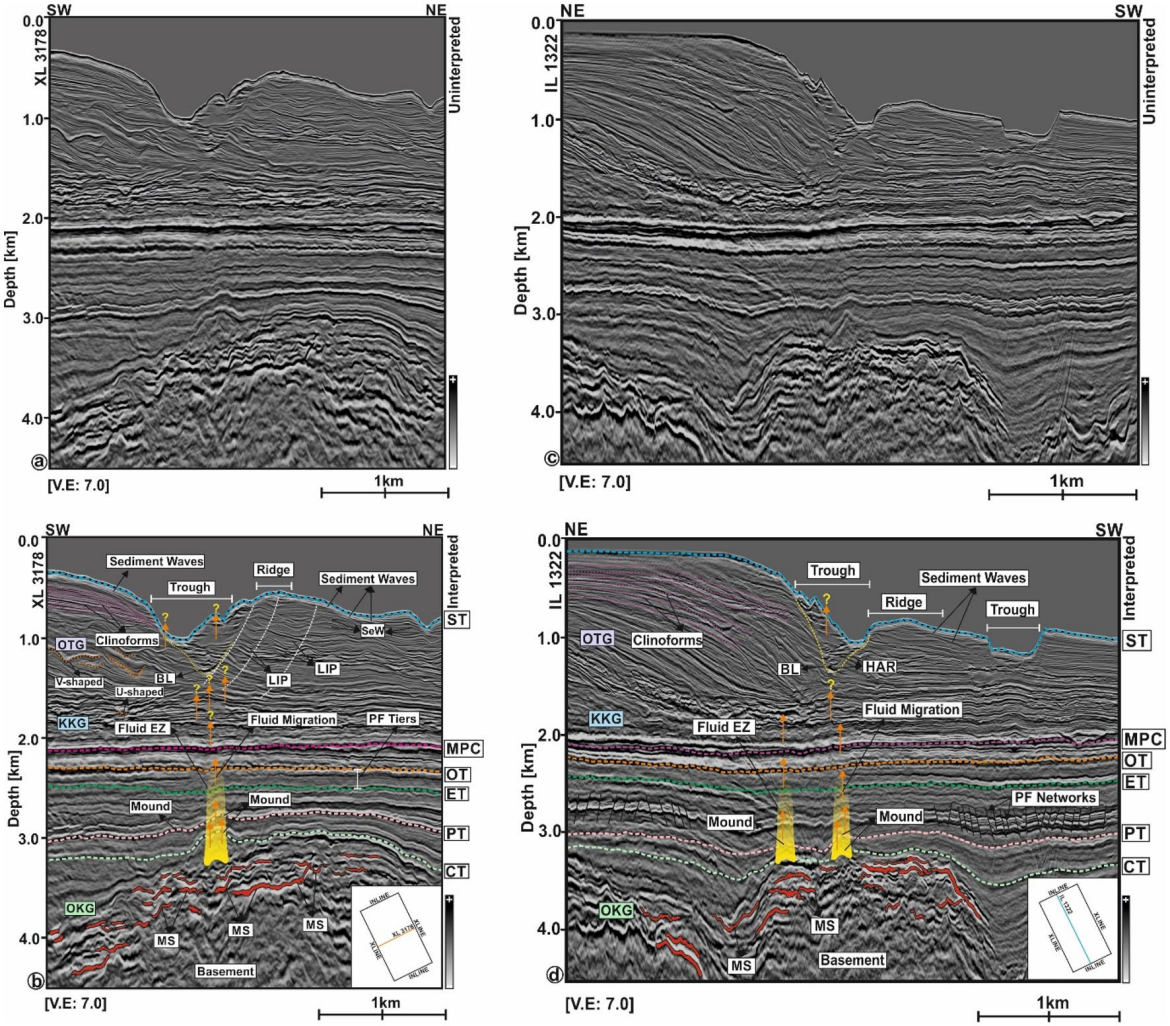
(**a**–**c**) Uninterpreted seismic lines XL 3178 and IL 1322 (**b**–**d**) corresponding interpretation on the subsurface structural framework of the Endurance 3D seismic survey. Subsurface strata mainly consists of three stratigraphy units: the Onekakara Group (OKG), the Kekenodon Group (KKG) and the Otakou Group (OTG). The zone of interest for this research is primarily focused on the seafloor and near seafloor strata (up to a depth of ~ 1200 m below the seafloor) into which fluids have migrated from the Cretaceous sediments through the KKG and the OTG. The seafloor is incised by the Waitaki Submarine Canyon, which is underlain by elongated clinoforms in its SW flank, basal lags at the canyon thalweg, clinoform packages, sediment waves, V- and U-shaped channel complexes and depressions over its northern banks. *MS* Magmatic Sill, *CT* Cretaceous Top, *PT* Paleocene Top, *ET* Eocene Top, *OT* Oligocene Top, *MPC* Marshall Paraconformity, *ST* Seabed Top, *SeW* Sediment Waves, *EZ* Escape Zone, *LIP* Laterally Inclined Package, *BL* Basal Lag, *PF* Polygonal Fault.

Seismic reflections within the Upper Cretaceous to Oligocene Onekakara Group are of moderate to high amplitude, continuous and wavy. Cretaceous and Paleocene strata show moderate-amplitude reflections and, locally, mounded structures (Fig. 6b–d). Reflections within these mounds are folded. Eocene and Oligocene strata are continuous and comprise multiple tiers of polygonal faults (Fig. 6b–d). The latter faults are associated with strata showing moderate amplitude, continuous reflections. Upwards, the sequence is topped by a regional seismic marker called the *Marshall Paraconformity*^27^, which separates the Onekakara and the Kekenodon groups. The Kekenodon Group contains strong wavy seismic reflections towards its western part, whereas seismic reflections are continuous and horizontal towards the east. The central part of this stratigraphic unit is the locus of several pull-up reflections (Fig. 6b–d).

Seismic reflections in the Otakou Group are moderate in amplitude (Fig. 6b–d). The western flank is characterised by the presence of several elongated clinoforms, named *Canterbury Drifts*^29^, in which reflections are inclined and sub-parallel. In the study area, these clinoforms are more prominent towards the SW and NE on the dip and strike seismic profiles in Fig. 6b–d. Apart from the latter clinoforms, stacked channel belts marked by V- and U-shaped incisions are observed.

The seafloor is dissected by the Waitaki Submarine Canyon and structured by alternating troughs and ridges. Laterally inclined packages (LIPs)^6^ are observed both on the NE and SW flanks of the canyon. These packages are made of stacked sigmoidal-shaped strata forming a thick sediment wedge (Fig. 6b–d). The canyon system is underlain by basal lags deposited in the canyon thalweg (Fig. 6b–d). The seafloor is surrounded by sediment waves in the east and western parts of the study area.

### Seismic character of near-seafloor and deeper strata

**Figure 7:**
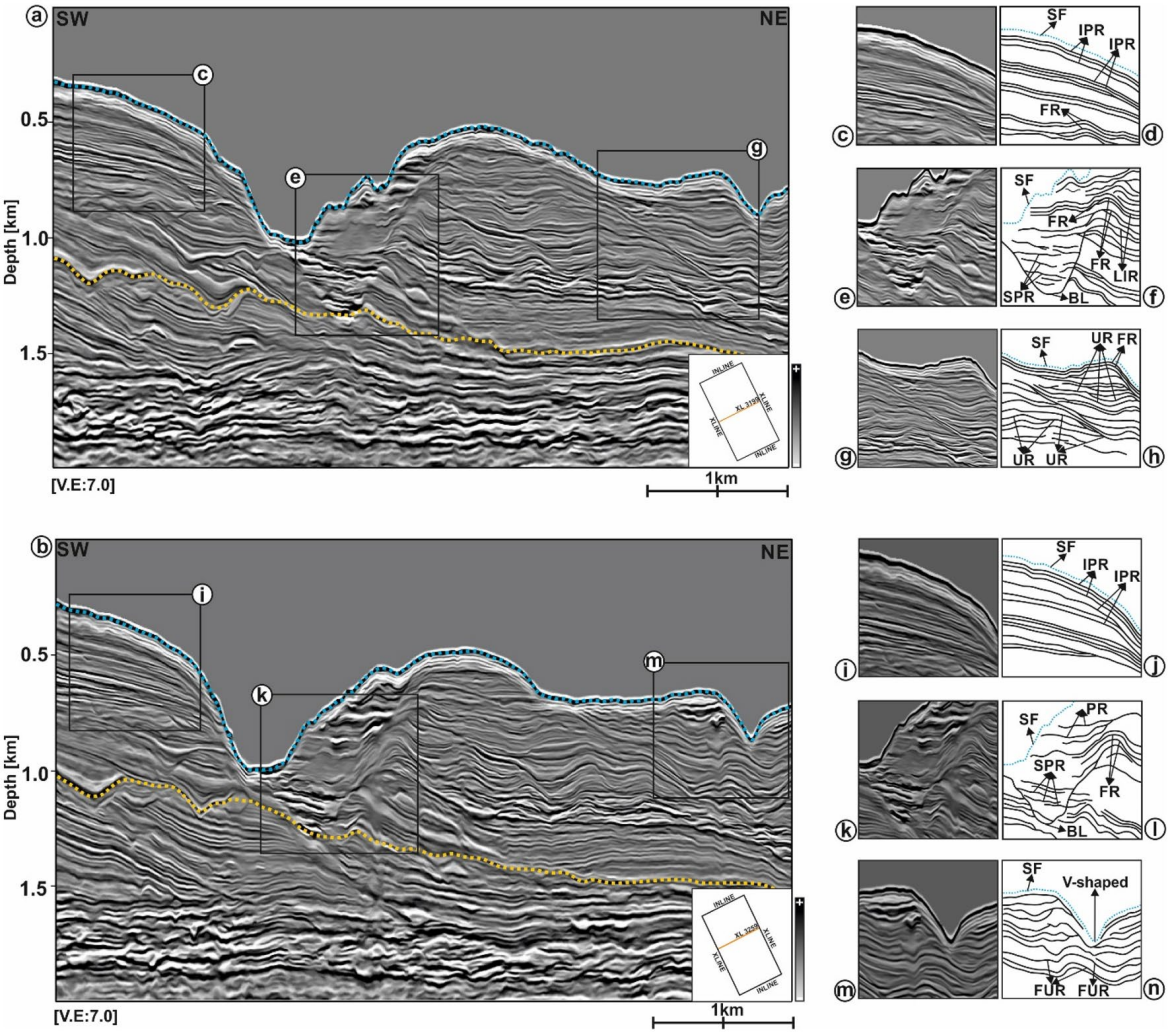
Seismic reflection character of the Waitaki Submarine Canyon as observed in lines (**a**) XL 3199 and (**b**) XL 3259 in the SW, central and NE parts of the study area. The seismic characters (**c**–**g,i**–**m**) are interpreted in the adjacent sketches (**d**–**h**,**j**–**n**) to understand their reflection geometries. The tops of seafloor and the near-seafloor strata are marked using blue and yellow dotted lines. *SF* Seafloor, *IPR* Inclined Parallel Reflections, *FR* Folded Reflections, *SPR* Stacked Parallel Reflections, *LIR* Laterally Inclined Reflections, *FUR* Folded Upward Reflections, *UR* Undulated Reflections, *PR* Parallel Reflections, *BL* Basal Lag.

The seafloor and strata up to a depth ~ 1200 m below the seafloor are wavy and reveal a variety of seismic-reflection patterns (Fig. 7a–n). In the SW sector of the study area, seismic reflections forming elongated clinoforms show moderate to low amplitude (Fig. 7a–d,i,j). Strata underlying these clinoforms are wavy, accompanied by moderate-amplitude folded reflections and several V- and U-shaped channels. In the centre of the study area, the seafloor is incised by the larger Waitaki Submarine Canyon and is clear, on selected seismic profiles, that the erosional surface below the canyon contains a basal lag in which reflections are sub-horizontal (Fig. 7a,b,e,f,k,l). This same basal lag is characterised by presenting high- to moderate-amplitude seismic reflections.

**Figure 8:**
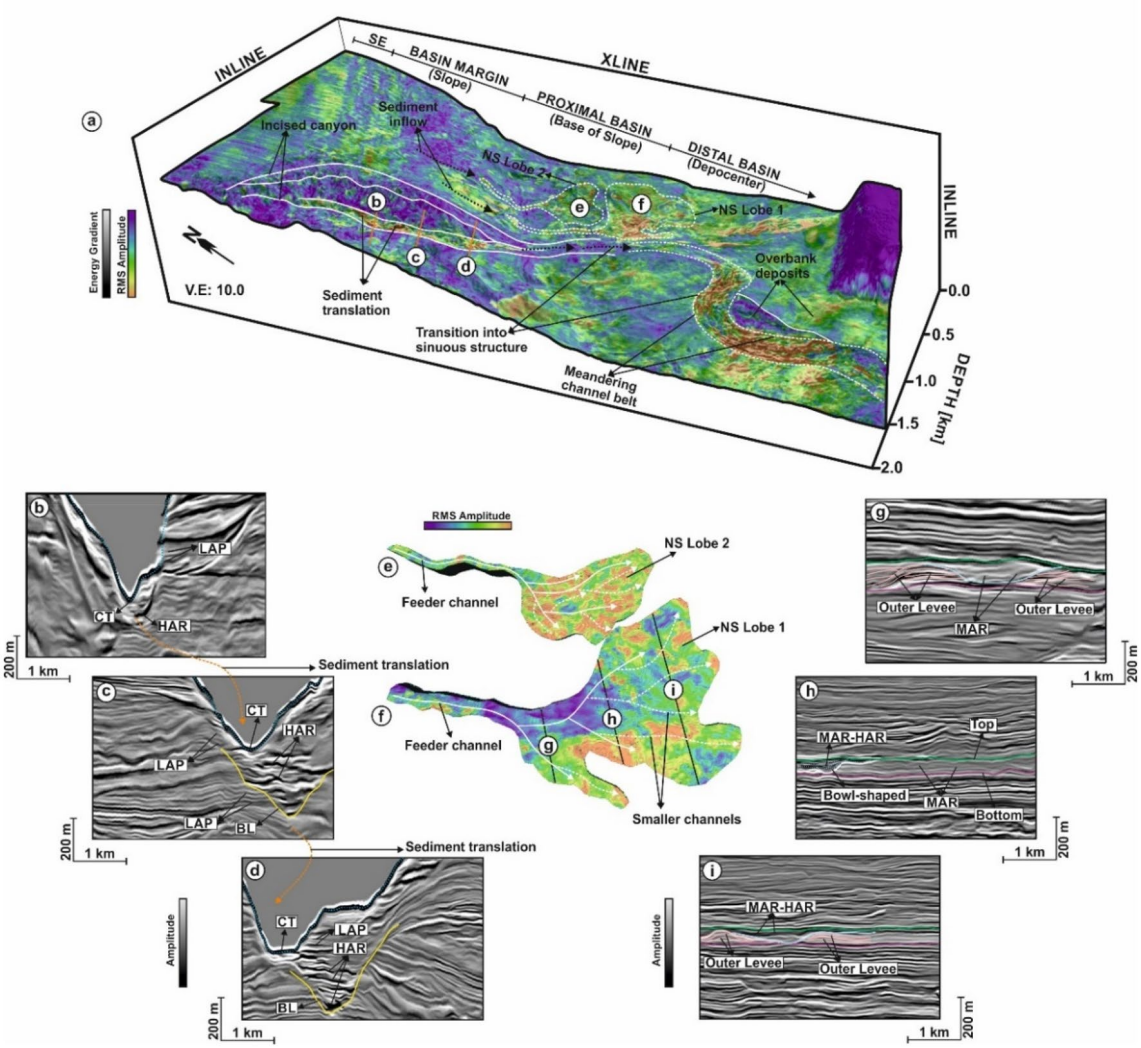
(**a**) TWT structure map of near-seafloor strata (~ 1200 m below the seabed top) overlain with RMS amplitude and energy gradient attributes. Different depositional elements of the turbidite system at the proximal and distal part of the basin are observed. These includes, the incised canyon, channel fills, lobes and over-bank deposits. Two distinct channel lobes namely NS Lobe 1 and NS Lobe 2 are observed on the NE part of the strata; (**b**–**d**) Seismic cross-sections through the channel fills below the canyon thalweg upon making transition from the basin margin (slope) to the proximal part of the basin (base of the slope). The canyon top is indicated using blue dotted line and the basal lag surface is delimited using a yellow line; (**e**–**i**) 3D structure of the channel lobes and seismic cross-sections crossing the lobe (NS Lobe 1 as an example). Internally the lobes are associated with smaller channels (marked using white dotted arrow) fed by the main channel source from the proximal area (marked using thick white arrow). The lobes interfinger in the distal area and are dominated by high amplitude reflections. In cross-sections, the top and bottom surface of the lobe is indicated using green and pink line and the levees are marked by cream lines. (*CT* Canyon Thalweg, *NS* Near seafloor, *LAP* Lateral Accretion Package, *HAR* High Amplitude Reflection, *BL* Basal Lab, *MAR* Moderate Amplitude Reflection, *SE* Shelf Edge). (The above maps are prepared using licensed version of Corel Draw v16 and OpendTect interpretation Suite v6.6.3).

High-amplitude reflections in the basal-lag deposits point out to the presence of coarse-grained sands and gravels^30^ with alternating moderate- to low-amplitude reflections, suggesting the accumulation of muddy sediment. Strata at a depth between 600 and 1500 m below the seafloor consist of a network of turbidite systems indicating different depositional environments. These elements include thalwegs that transition into distinct sediment lobes to the NE (Fig. 8a,e–f). The root-mean-square attribute map in Fig. 8a shows turbidite elements with moderate- to high-amplitudes, a character indicating the presence of sand-prone sediments transported basinwards through the canyon (Fig. 8a,b–d). Sediment lobes interfinger with several smaller channels (Fig. 8a,e,f,g–i). Such an interpretation suggests these sediment-lobe complexes form a series of interfingering sediment bodies that are chiefly composed of sand (Fig. 8a).

**Figure 9:**
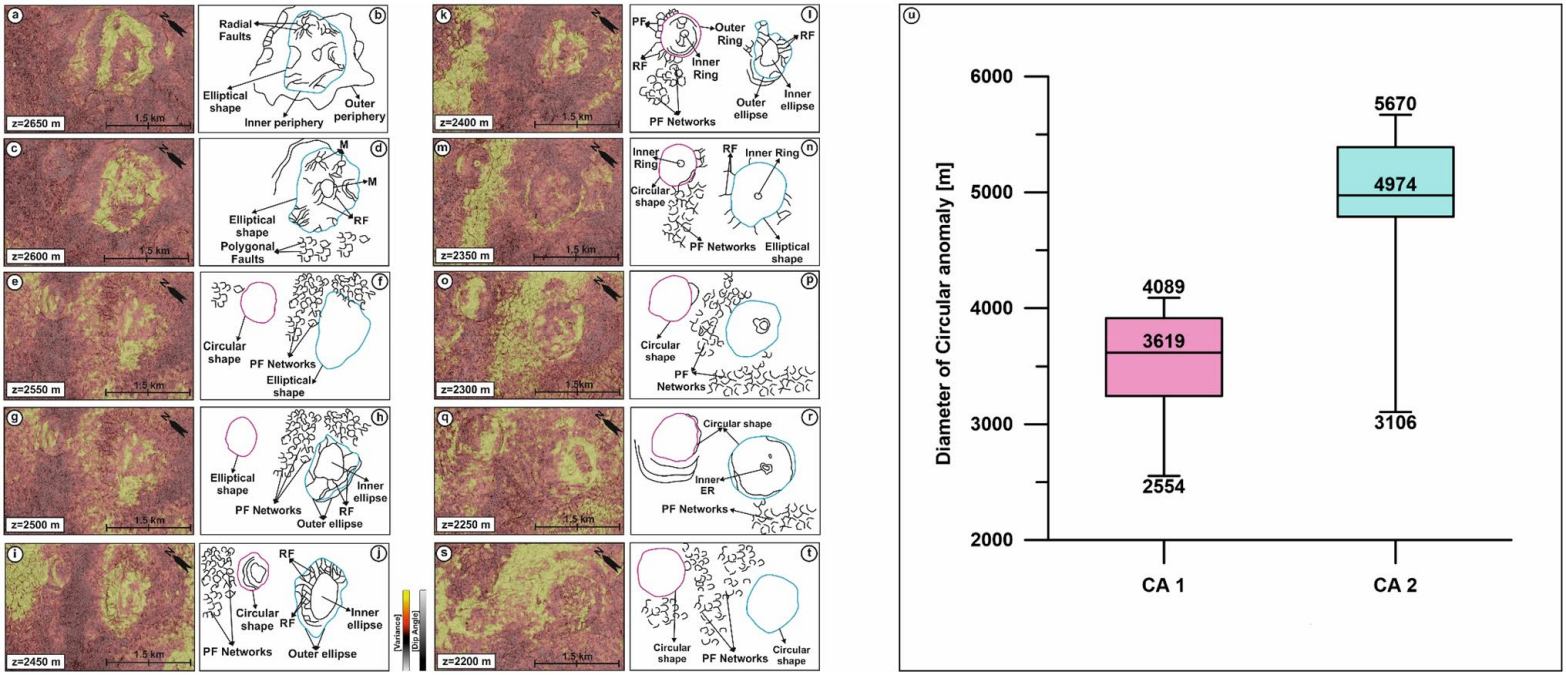
(**a**–**t**) Circular Anomalies (CA 1 in the blue oval and CA 2 in the red oval) and the corresponding interpretation sketches observed at depths ~ 2650–2200 m through the Eocene to the MPC intervals. The depth maps are prepared co-rendering the variance and dip-angle attributes; (**u**) Box-whisker pot showing the variation in the diameter for the observed circular anomalies i.e., CA 1 and CA 2 respectively.

Strata at a depth of ~ 3000–3500 m below the seafloor, within the Cretaceous and Paleocene intervals, reveal a series of mounded structures (Fig. 6a–d). Two distinct circular anomalies resembling craters (CA 1 and CA 2; Fig. 9a–t) are observed above these elevated mounds. These anomalies occur in Eocene strata at a depth between 2200 and 2650 m below the seafloor. Crater CA 1 is characterised by an elliptical geometry at depths between 2650 and 2400 m. The outer boundary of CA 1 possess an elongated lobate structure, whereas its inner boundary resembles an elliptical shape (Fig. 9a–d). This elliptical structure contains several circular mounds associated with radial faults (Fig. 9a–d). Towards the SW, crater CA 1 is surrounded by networks of polygonal faults (Fig. 9c,d). At a depth of 2500–2550 m below the seafloor these same polygonal fault networks surround the eastern and western regions of the CA 1 (Fig. 9e–h).

Crater CA 2 has a circular geometry and occurs at a depth of 2550 m below the seafloor (Fig. 9e,f). Such a circular geometry continues upwards to a depth 2200 m (Fig. 9e–t). The irregular elliptical geometry of CA 1 continues to a water depth of 2350 m, which then achieves a circular shape at a water depth of 2200–2300 m (Fig. 9g–t). Throughout these depth intervals, circular anomalies in CA 1 and 2 are surrounded by polygonal (PFs) and radial faults (RFs). The polygonal faults occur in tiers within Eocene–Oligocene strata. Craters CA 1 and CA 2 reveal an average diameter of ~ 3.619 km and ~ 4.0 km, as recorded by the box-whisker plot in Fig. 9u.

## Discussion

### Continental margin architecture as a function of submarine canyon evolution

The Canterbury Basin is characterised by three different stratigraphic units reflecting multiple transgressive–regressive cycles: (a) the transgressive Onekakara Group, (b) the Kekenodon Group reflecting a highstand in sea level, and (c) the regressive Otakou Group (Fig. 6a–d). The basin witnessed major changes in regional tectonics from late Oligocene to the early Miocene when of the initiation of strike-slip movements in the Alpine fault system^31^, uplift of the Southern Alps, and subsequent deposition of a regressive megasequence^26,29^. This resulted in the transport of terrigenous sediment basinwards, incising the Otago Shelf. Repeated incision and erosion of the continental shelf and slope, generated a network of V-shaped submarine canyons^3,5^. Thus, an interplay of tectonic uplift in the Southern Alps, supply of fluvial sediments from different sources, and the effect of marine currents associated with the Southland Front^18,29^ controlled the sedimentary processes associated with the formation of the interpreted submarine channels. In addition, Milankovitch cycles controlled sea level fluctuations (spanning periods of time of 20–120 ka), and subsequently the distribution of architecture of sediments deposited in the Canterbury Basin during the Late Quaternary^18^.

The TWT structure map of the seafloor, obtained from both seismic and bathymetric data, reveal that the shelf edge is sharply incised by steep canyons and gullies (Figs. 3, 4). These features resemble a Type I canyon following the earlier classification scheme^5^. The geological processes that shape these types of canyon include erosion, and the effect of turbidity currents and high-volume mass flows^2,3,5^. Moreover, it is observed that canyon morphology was maintained by these multiple depositional processes—turbidity currents resulted in the deposition of lateral accretion packages on the banks of the Waitaki Submarine Canyon (Figs. 6, 8). Several studies have considered that turbidite currents lack the ability to erode the continental shelf and slope as they are mostly associated with thick, dilute, muddy and sluggish turbidity flows^3,5,32^. Erosional currents are more vigorous in nature and result in the deposition of high amplitude reflections (or HARs *sensu*^3,30^), as observed in the study area below the canyon thalweg and its basal lags (Figs. 6, 8b–d). Furthermore, inter- and intra-canyon geometries are controlled by the deposition of laterally inclined packages, which has led to progradation of a thick sediment wedge (Fig. 6). Remarkable continuity and consistent amplitude responses within these zones suggest that inter-and-intra canyon strata are controlled by the pre-existing seafloor topography and may not be the sole result of current activity.

### Distribution of porous, sand-rich sediment in the Waikato Submarine Canyon

The seafloor towards the SE coast of South Island is characterized by a narrow and shallow continental shelf (Fig. 1b,d), beneath of which lies the thin continental crust of the Campbell Plateau^20^. The Waitaki Submarine Canyon acted as a transfer zone for transporting sediments from the shelf to the Bounty Trough^19^. At its lower reach (i.e., towards base of the slope) the canyon pathways connect to the North Bounty channel (Fig. 1b), ultimately merging with the south and central channel to form the Bounty Channel at a distance approximately 200 km seawards from the shelf edge.

Our structurally-conditioned 3D seismic data show that continuous transport of sediment through shelf-incised submarine canyons resulted in the deposition of large volumes of turbidites further downslope in the form of channel overbanks, meandering channel belts, channel-fill deposits and sediment lobes in the NE part of the study area (Fig. 8). The SW region preserves the sinuous channel of the Waitaki Submarine Canyon and overbank deposits (Fig. 8). These basin fill deposits form the key elements of turbidite systems associated with submarine canyons^3,33^. Some of the noted examples of such canyon-channel systems includes the Mississippi Delta and Canyon^34^; the Hudson Canyon^35^ and the Swatch of no Ground in the Bay of Bengal^36^.

The erosional surface below the thalweg of the Waitaki Submarine Canyon preserves high-amplitude reflections, which are here interpreted to reflect the presence of coarse-grained (essentially sandy) sediment. Furthermore, lateral accretionary packages are observed on the sidewalls of the canyon (Fig. 8). This suggests the concavity of the Waitaki Submarine Canyon to be controlled by two sets of turbidity currents namely (a) weakly erosive currents and (b) highly vigorous erosive currents that are mostly associated with high-amplitude reflections. Sediments downslope are mostly sandy, reflecting with high shear rates at their base, and deposited as a result of high density turbidity currents, when compared to their upslope counterparts. Thus, high-low density turbidity currents associated with variations in shear stress (*sensu*^37^) led to the erosion of mud-rich cohesive sediments, thereby transporting sandy sediments downslope. These sand-dominated deposits were further preserved within the lobe complexes and sinuous channel belts in the NE and SW parts of the study area. This is clearly shown by the high-amplitude contrasts within the turbidite elements of these two latter areas (Fig. 8a,e,f).

### Channel-fill deposits and lobes: are they capable of focusing fluid in deep-offshore basins?

**Figure 10:**
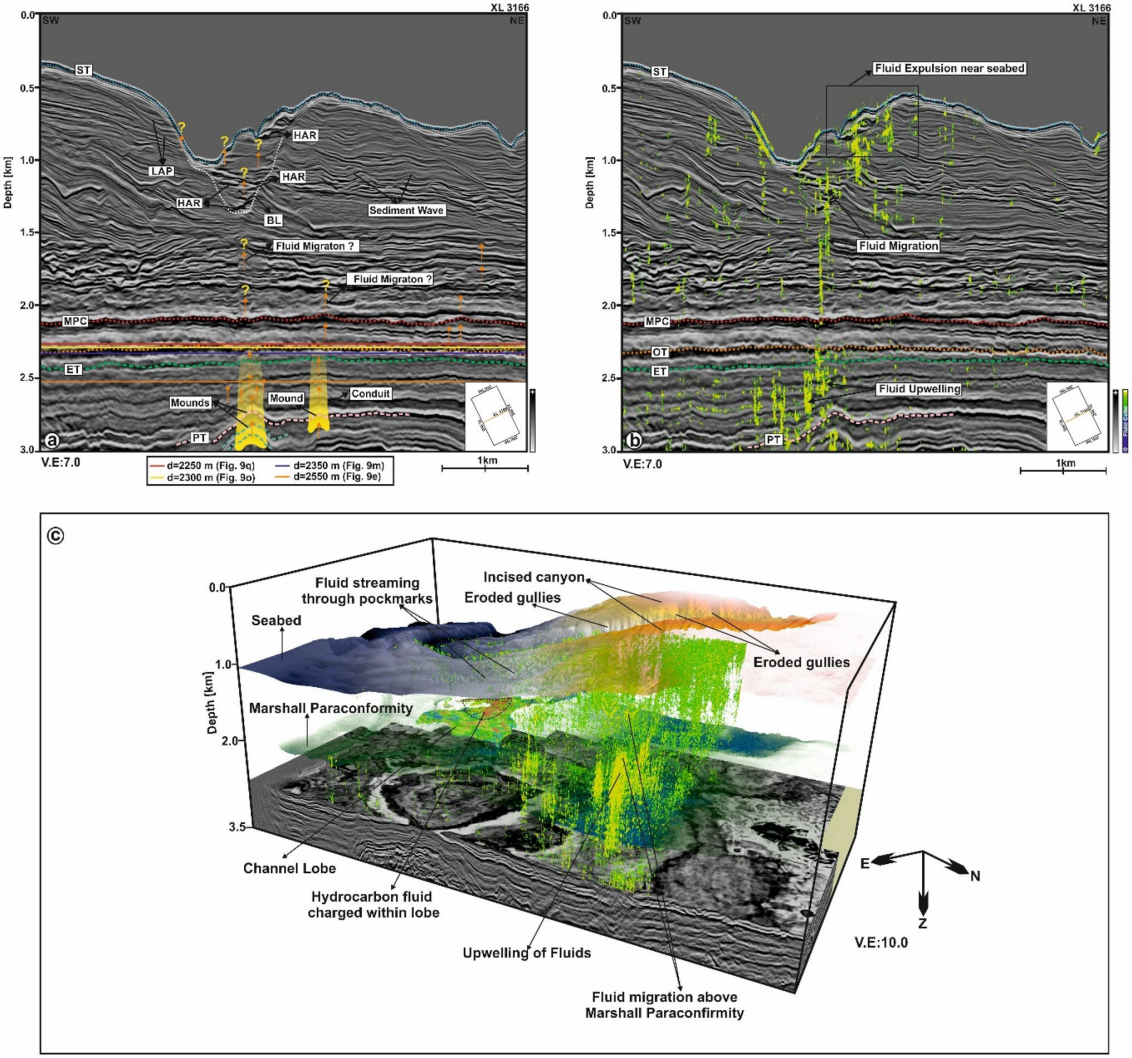
(**a**) Interpreted seismic line XL 3166 revealing fluid seepage from Cretaceous to Palaeocene, Eocene and Oligocene strata. Fluid is proposed to seep through different mounded structures observed within these intervals. However, above the MPC fluid seepage activity is not clear. The presence of high-amplitude seismic reflections within the basal lag bellow the canyon thalweg and pull-up reflections observed in Fig. 6 provides an hint for fluid migration through shallow strata all the way to the seafloor; (**b**) Fluid cube meta-attribute intelligently strengthens the proposition in (**a**) by presenting a realistic interpretation of fluid migration from deeper sediments into the shallower intervals i.e., through the canyon walls all the way to seafloor; (**c**) 3D view of fluid expulsion from the Paleogene sediments through the Neogene intervals into the present day seafloor. The 3D visualisation reveals the presence of shallow reservoirs i.e., channels and lobe complexes that acts as repository for the migrated fluids. (*LAP* lateral accretion package, *HAR* high amplitude reflection, *BL* Basal Lab, *MPC* Marshall Paraconformity, *PT* Paleocene Top, *OT* Oligocene Top, *ET* Eocene Top, *ST* Seabed Top). (The above maps are prepared using licensed version of Corel Draw v16 and OpendTect interpretation Suite v6.6.3).

Deposits within the sediment lobes are high permeable zones that host significant amount of fluids migrating from deeper strata^23^. Tectonic movements during the Late Miocene to Recent, followed by incision of the Waitaki Submarine Canyon, generated the necessary conditions to breach the seal intervals covering these reservoirs, thereby allowing the fluids to migrate through overlying sediments and feed the mapped seafloor pockmarks (Fig. 10a,b).

Early research has demonstrated that pockmarks indicate past or ongoing fluid expulsion processes in which the dissociated fluid is predominantly related to hydrocarbon migration^38^. In this regard, elongated or elliptical pockmarks over seafloor are more active in streaming fluids (gas and liquids) through sediments, and the evolution of such depressions is commonly related to escape of hydrocarbon fluids from underlying fluid sources^9,38^.

Previous research has also shown that seafloor depressions in the Otago shelf and the surrounding zones are the result of modifications by submarine currents of the Southland Front, which significantly altered the size and alignment of these structures^21^. These results were more recently rebuked by proof that recent and past fluid flow in the Canterbury Basin originates from Cretaceous strata^39^. In this work, it is observed that the mapped pockmarks are clustered at a water depth of 500–900 m, with depressions varying in depth from 12 to 55 m (Fig. 4a–c). These pockmarks distinctly show two different morphologies, i.e., circular and elliptical or elongated, in which the most abundant are the elongated depressions occurring over the northern banks of the Waitaki Submarine Canyon (Fig. 8). We infer that fluid seepage at the seafloor, including active structures feeding hydrocarbon-rich fluids, formed the elliptical or elongated pockmarks mapped in this work (Fig. 4a–c).

To understand the story of fluid migration in the study area, an attempt has been made to employ machine tools to our dataset. The machine learning technique uses an artificial neural network to design a hybrid attribute called as the Fluid Cube meta-attribute that captures subsurface fluid flow events from the 3D seismic data (Fig. 10b). This has been successfully applied in other prospects by^40,41^. The meta-attribute distinctly confirms the presence of fluids sourced from the Cretaceous sediments and migrating through the Eocene and Oligocene strata. It also reveals fluid seepage above the *Marshall Paraconformity*; migrated fluids are trapped within the channel lobes and fans, which acts as shallow reservoirs. In addition, the fluids use the eroded gullies of the canyon as pathways to migrate onto the seafloor. Our 3D Fluid Cube efficiently strengthens these interpretations, stressing the importance of fluid migration through Paleogene and Neogene sediments in the Canterbury Basin (Fig. 10c). It also demarcates the presence of shallow hydrocarbon and potential geothermal reservoirs in the NE part of the study area.

## Conclusions

A high-resolution 3D depth-converted seismic volume allowed us to investigate the structural morphology of a submarine canyon, the Waitaki Submarine Canyon, and the role of this canyon system in focusing fluid flow along the Otago shelf, SE New Zealand. The main conclusions of this study are as follows:

1. The Waitaki Submarine Canyon is a Type I canyon, incised at the shelf-break and reveals a V-shaped cross-section near the continental shelf. It changes into a U-shaped cross-section on the continental slope. The canyon is located in water depths ranging from ~ 400to 1200 m and shows a common NW–SE orientation.
2. The head of the canyon is sharp, concave, and incises both the upper continental shelf and the shelf break. It dips at ~ 10°–20° on the upper continental slope, gradually widening downslope. The gullied slope succession of the canyon is associated with several eroded ridges.
3. The morphology and development of the canyon is significantly influenced by sediment supplied from hinterland sources, the Southland Current and regional tectonic activity.
4. The presence of channel-fill deposits and lobes near the seafloor hints at the occurrence of shallow hydrocarbon reservoirs in the study area.
5. Focused fluid flow is observed throughout the study area, whereby fluid migrates from deep Cretaceous sediments through mounded structures observed in Palaeocene-Oligocene strata. It is funnelled in the shallow channel-fill deposits and lobes, being lost (seeped out) through the Waitaki Submarine Canyon.
6. The new reprocessed Fluid Cube meta-attribute captures this geological scenario with great accuracy and resolution from the depth-migrated seismic volume.

This study reveals the presence of shallow hydrocarbon reservoirs underneath the Waitaki Submarine Canyon that may be potential exploration targets along the continental margin of New Zealand. Moreover, it is an important case study documenting the geological processes associated with shelf-slope depositional systems and associated geohazards.

## Data and methods

The data used in this study include a depth migrated 3D seismic volume comprising 810 inlines and 2967 crosslines, and latest gridded bathymetric data^42^ (Figs. 1, 3). The seismic volume was acquired by the R/V Polarcus Alima^43^ in December 2013 and covers an area of 650 km^2^. Water depth in the surveyed region ranges from 100 to 1600 m.

The primary goal of the seismic survey was to provide accurate images of submarine canyons and underlying structures, identify shallow submarine channels and resolve velocity variations due to local geology such as shallow gas seeps, limestone intervals, and deeper volcanic sills^44^. The recognition of the Waitaki Submarine Canyon is based on the criteria proposed by^2^ in which submarine canyons are ‘defined as steep walled, sinuous valleys with V-shaped cross-sections, axes slopping outwards’.

The interpreted seismic data were processed to a bin size of 25.0 m by 12.5 m, a record length of 8.2 s, and to a 2 ms sampling rate^43^. Data processing also included noise attenuation, multiple elimination, broadband processing, regularisation, velocity modelling, pre-stack depth migration, gather flattening, demultiple followed by stacking and post-stack processing. The length of the depth migrated seismic cube is 8 km.

This work uses a newly prepared depth-migrated seismic cube of the Endurance seismic survey procured for academic research by the New Zealand Petroleum and Minerals (NZP&M). This depth-migrated seismic volume was considered the best dataset for this study as one can directly and accurately compute morphometric parameters (thickness, length, width, area etc.) rather than having to use velocity data for time-depth conversion. The volume is displayed using SEG’s American polarity convention, whereby an increase in acoustic impedance is represented by a positive-amplitude black reflection.

The bathymetric data used in this study comprise the most recent bathymetric grid developed by the Nippon Foundation-GEBCO. The grid is a continuous, global terrain model for ocean and land with a spatial resolution of 15 arc seconds^45^. The gridded data set is an amalgamation of land topography with measured and estimated seafloor bathymetry^42^. The bathymetry data were initially examined to map the geometry of the Waitaki Submarine Canyon (Fig. 3a–i). The depth-migrated seismic cube was then used for a detailed interpretation of the seafloor, associated seafloor depressions and underlying structures.

Prior to seismic interpretation, the depth-migrated seismic cube was structurally conditioned using a structure-oriented filter (SOF), named herein as dip-steered median filter (DSMF) so as to improve the resolution of geologic structures. This filter has been successfully applied to different offshore prospects^42,43,46,47^. The DSMF applies median statistics over seismic amplitudes following the stored seismic reflection dips and azimuth at every sample location from a pre-processed steering cube, which is prepared through a dip-steering process based on the phase-based dip algorithm^48^. In this work, the filtering of the seismic cube was performed using a 3 × 3 median filtering step-out. The key objective behind this filtering technique is to differentiate between the dip-azimuth of the seismic reflectors and overlying noise, therefore removing random noise from the data while preserving the amplitudes and enhancing the lateral continuity of the seismic events. Seismic interpretation involved mapping of the seafloor top from seismic data and morphometric analysis of the features observed over the seafloor. Furthermore, a machine learning approach was used to visualize fluid migration through the seafloor. This approach has successfully been previously applied by authors^42,43^ in the Taranaki and Canterbury Basins, offshore New Zealand.

Seventy-five (75) seafloor depressions, herein referred to as pockmarks, were identified in this work. Their morphometric properties such as depth, diameter and flank gradients were interpreted from detailed seafloor maps and seismic profiles. The parameters above were used to ascertain the morphometry of the mapped pockmarks. Seismic attribute slices at different depths below the seafloor were used to interpret sub-seafloor structures.

## Data Availability

The data, used in applying this approach, was procured from the New Zealand Petroleum and Minerals, Ministry of Business, Innovation and Employment, New Zealand, [https://www.nzpam.govt.nz/] under certain restrictions and guidelines, and thus the data are not publicly available. However, the data can be procured for research with reasonable request and undertaking, and permission by New Zealand Petroleum and Minerals, Ministry of Business, Innovation and Employment, New Zealand [https://www.nzpam.govt.nz/].
